# Managing Encephalopathy in the Outpatient Setting

**DOI:** 10.5005/jp-journals-10018-1211

**Published:** 2017-05-05

**Authors:** Tarana Gupta, Sahaj Rathi, Radha K Dhiman

**Affiliations:** 1 Department of Medicine, Pandit Bhagwat Dayal Sharma Post Graduate Institute of Medical Sciences, Rohtak, Haryana, India; 2 Department of Hepatology, Postgraduate Institute of Medical Education & Research, Chandigarh, India

**Keywords:** Cirrhosis, Hepatic encephalopathy, Minimal hepatic encephalopathy, Neurocognitive testing.

## Abstract

In cirrhosis of liver, hepatic encephalopathy (HE) has an important impact on health-related quality of life. It is important to define whether HE is episodic, recurrent, or persistent; types A, B, or C; overt HE or covert HE; and spontaneous or precipitated. The overt HE is clinically evident and needs hospitalization. Nonabsorbable disaccharides, rifaximin, and probiotics are proven to be useful in the treatment of overt HE. Covert HE includes both minimal HE and grade I HE. It is not apparent on routine clinical examination. Presence of poor work productivity, increased accidental injuries on complex machinery and driving, etc., raise the suspicion of cognitive dysfunction. Specialized neurocognitive testing like psychometric HE, computerized tests like critical flicker frequency tests, inhibitory control tests, Stroop encephalopathy tests, and electroencephalography are needed to diagnose overt HE. Various studies have shown lactulose and rifaximin to be useful in overt HE. However, presence of persistent and recurrent HE in cirrhosis is an indication for liver transplant. Lactulose is effective both in improving reversal of minimal HE and in reducing the risk of development of overt HE.

**How to cite this article:** Gupta T, Rathi S, Dhiman RK. Managing Encephalopathy in the Outpatient Setting. Euroasian J Hepato-Gastroenterol 2017;7(1):48-54.

## INTRODUCTION

Hepatic encephalopathy (HE) is a frequent and one of the most devastating complications of cirrhosis of liver and acute hepatitis. It is compounded by its tendency toward recurrence and persistence of cognitive decline with each episode. This progressive cognitive decline becomes really challenging and exhaustive to manage. The lack of consistency and universally accepted standards in defining HE have caused the investigators to include a wide spectrum of cognitive dysfunction into HE; now terms like covert HE and overt HE have been introduced.^1^ Over the years, with the advent of new therapies and drugs, there is significant advancement in the management of HE with better outcomes. The latest studies support the evidence that encephalopathy in cirrhosis is multifactorial, relating to ammonia metabolism, portosystemic shunting, systemic inflammation, and neuroinflammation. Emerging role of antibiotics and probiotics in addition to traditional lactulose therapy also emphasizes this fact.^2^

## DEFINITION OF HE

Hepatic encephalopathy is a brain dysfunction caused by liver dysfunction and/or portosystemic shunting, which manifests as a wide spectrum of neurological or psychiatric abnormalities ranging from subclinical alterations to coma.^1^

## CLASSIFICATION OF HE

The HE in cirrhosis needs to be classified accurately for selection of appropriate therapy. The following four points need to be addressed in each case.

1. Underlying disease
  - Type A: Caused by acute liver failure
  - Type B: Caused by portosystemic shunting
  - Type C: Caused by decompensated cirrhosis and liver cell dysfunction
2. Time course and duration
  - Episodic HE: Occurring once in more than 6 months
  - Recurrent HE: Episodes occurring in less than 6 months
  - Persistent HE: In between the episodes, there is persistence of behavioral disturbances
3. Precipitating factors
  - Spontaneous: No precipitating event
  - Precipitated: Precipitant event(s) such as infection, constipation, upper gastrointestinal bleed, electrolyte disturbances, diuretic overdose, etc., are present.
4. Severity of manifestations
  - Covert HE: It includes minimal and West Haven grade I HE
  - Overt HE: It includes West Haven grade II-IV HE

## BURDEN OF DISEASE

Due to subjective assessment of symptoms and heterogeneity of definitions in various studies, there is variation in reported prevalence of HE. However, overt HE is present in 16 to 21% in decompensated cirrhosis and 10 to 50% in posttransjugular intrahepatic protosystemic shunting (TIPSS).^3^ Overall, 20 to 80% of patients with cirrhosis of liver have either minimal HE or covert HE at some point during the course of their illness.^4^ Sharma et al^5^ found a minimal hepatic encephalopathy (MHE) prevalence of 53% in cirrhosis with 43% in Child A, 59% in Child B, and 62% in Child C patients. Patients with a single episode of overt HE were found to have 1-year cumulative risk of 40% for recurrent overt HE, and patients with recurrent overt HE had a cumulative risk of 40% to develop another episode of overt HE within 6 months despite being on lactulose therapy.^6^

## CLINICAL PRESENTATIONS

### Overt HE

Overt HE may present as lethargy or apathy, disorienta-tion for time, increased daytime sleepiness with altered sleep-wake cycle, obvious personality change, inappropriate behavior, dyspraxia or flapping tremor in the initial stage to somnolence, semistuporous, and confused state with gross disorientation. On physical examination, patients may have ataxia, increased deep tendon reflexes, and Babinski’s sign. The West Haven Criteria are gold standards for the diagnosis of overt HE. In patients with altered consciousness, the Glasgow coma scale is used for clinical assessment.

### Covert HE

Covert HE consists of minimal HE and grade I HE. It may be completely asymptomatic or may have trivial symptoms. The clinical suspicion is raised in patients with symptoms of euphoria, anxiety, shortened attention span, and impairment of addition or subtraction ability. Patients have history of repeated accidents, traffic rule violations, and driving disabilities resulting from poor attention span, impaired visuomotor coordination, and increased reaction time.^7^ Cognitive function decline and poor work productivity and performance may lead to loss of job and position. Patients with grade I HE have higher mortality than patients without HE.^8^

## DIAGNOSIS OF MHE

The diagnosis of overt HE is made based on history and clinical examination, whereas MHE may be apparent only on specific neurocognitive testing (Table 1). The presence of subtle clinical features raises the suspicion and these patients should be assessed with mini mental state examination (MMSE). An MMSE score >24 practically rules out the possibility for overt cognitive impairment, and these patients may be subjected to neurocognitive testing.

The psychometric hepatic encephalopathy score (PHES) consists of five tests.^9^ These are number connection tests A and B, line tracing test, serial dotting test, and digit symbol test. For illiterate people, the number connection test may be replaced by figure connection test.^10^ The PHES score may be adjusted for age and education of the concerned patient. The PHES has been validated in many studies and is considered the gold standard in diagnosis of MHE. The drawbacks of PHES are that it is time consuming, needs learning time, and is dependent on age and education.

The newer computerized tests like inhibitory control test (ICT), Stroop test, continuous reaction test, and scan test are easy, quick, and require minimal training. However, they are applicable in only high-functioning patients. The drawback is that they are not validated as PHES score.^11^

In MHE, the electroencephalography (EEG) can reveal slowing of brain activity. The EEG does not require learning abilities and patient cooperation. However, it is expensive and time-consuming.

The critical flicker frequency (CFF) test establishes the ability of brain to detect the flickering of light, which indirectly points toward brain cortical activity. This is an easy, cost-effective test, which is unlike PHES score independent of education.^12^ Romero-Gomez et al^13^ found that CFF <38 Hz was predictive for development of further episodes of overt HE in patients of cirrhosis.

The Stroop test assesses the activity of brain to detect color of the word when the semantic meaning of the given word is a different color. It assesses the cognitive function and is available as smartphone application.^14^ Allampati et al^15^ showed good correlation of Stroop encephalopathy application with PHES score. The ICT is another test for diagnosis of MHE, and various studies have shown it to be important for prediction of overt HE.^16^

**Table 1:** **Table 1:** Tests for diagnosis of minimal HE

*Test*		*Description*		*Reliability*		*Advantages*		*Disadvantages*	
*Paper-Pencil* PHES		5 paper and pencil tests number connection tests A and B; digit symbol; line tracing; serial dotting tests		Sensitivity: 96% Specificity: 100%		• Extensively validated		• Learning effect • Time consuming • Good neuromuscular control needed	
Repeatable battery for the assessment of neuropsychological status		Paper pencil battery testing 2 domains, cortical and subcortical		Good		• Has US reference data		• Copyrighted • Requires psychologist	
*Computerized* Inhibition control test		Presentation of letters at 500-ms intervals. Patients instructed to respond only when X and Y are alternating		Sensitivity: 87% Specificity: 77%		• Validated • Does not require psychologist		• Requires cooperative patients • Requires practice session	
Stroop encephalo application		Identification of the color of symbols or text presented, while the word names a different color		Sensitivity: >70% Specificity: 90%		• Easy and quick • Reliable • Freely available		• Cannot be performed in color-blind	
Scan test		Computerized digit recognizing task measuring the reaction times and errors		Mortality Hazard ratio: 2.4 (95% confidence interval 1.1-5.3)		• Reliable, predicts mortality		• Need practice sessions, knowledge of computer	
*Neurophysiological* Electroencephalogram and/ or evoked potentials		Can detect changes in cerebral activity across the spectrum of HE		Sensitivity: 43-100%		• No learning effect		• Needs neurologist • Expensive and labor intensiv	
Critical flicker frequency		Highest frequency at which the flicker of a light source can be detected, above which light is perceived to be continuous		Sensitivity: 40-100% Specificity: 91%		• Simple and reliable • Uninfluenced by age, education		• Requires highly functioning patients, binocular vision, absence of red-green color blindness	
Continuous reaction time		Repeated registration of the motor reaction time to an auditory stimulus		-		• Quick		• Requires good hearing	

Modified from Rathi and Dhiman^31^

The International Society for Hepatic Encephalopathy and Nitrogen Metabolism (ISHEN) recommends that two different tests should be used to diagnose MHE, which includes one of the tests, which is widely accepted to serve as the comparator.^17^

The upcoming modalities for diagnosis of cognitive dysfunction in cirrhosis are biomarkers and newer imaging tools. The S100(3 is an astrocyte protein produced after brain injury and found to be elevated in cirrhosis with MHE/overt HE as compared with healthy subjects.^18^ The lipid peroxidation and increased oxidative stress with synergistic effects in presence of hyperammonemia have also been implicated in pathogenesis of MHE. Gimenez-Garzo et al^19^ found correlation of malondialdehyde and 3-nitrotyrosine activity with cognitive decline in cirrhosis. The newer imaging modalities like diffusion tensor imaging and voxel-based morphometry have been used for detecting MHE; however, the data are limited and need further validation.

## TREATMENT OF HE IN THE OUTPATIENT SETTING

### Overt HE

The overt HE includes HE grades II to IV. The primary prophylaxis of overt HE is not routinely indicated except in cirrhosis with high risk to encephalopathy. The overt HE is most commonly precipitated due to infection, upper gastrointestinal bleed, electrolyte disturbances, constipation, etc., and treating the specific cause with antibiotics, bleed control, and correction of electrolytes and laxatives improves the encephalopathy. The spontaneous or precipitated overt HE needs to be treated with specific antihepatic coma measures. These are as described in Table 2 and Flow Chart 1.

Nonabsorbable Disaccharides

The lactulose is the mainstay of HE therapy. The lactulose is a synthetic disaccharide, which is undigested in small intestine and reaches the colon, where it is fermented. On fermentation, it acidifies the colonic environment that supports the growth of beneficial microflora. All these effects lead to decreased ammonia production and absorption. Also, by laxative effect, it decreases the ammonia. Therefore, there is reduction in blood ammonia levels and improvement in cognitive function. It is used for treatment of HE and for secondary prophylaxis of HE. The lactitol is a newer substance, which has been used in some studies. The nausea and bloating sensation are some side effects. The dose is 25 mL every 12 hourly until two to three soft stools occur. The dose can be titrated as per patient requirement. A systematic meta-analysis has shown nonabsorbable disaccharides to be useful in the treatment and prevention of HE.^20^ Lactulose is useful in the treatment of HE. Studies have established its role in primary as well as secondary prophylaxis of overt HE.

**Table 2:** **Table 2:** Agents used in outpatient treatment of HE

				*Role*							
*Therapeutic modality*		*Mechanism*		*Covert HE*		*Secondary prophylaxis of overt HE*		*Primary Prophylaxis of overt HE*		*Comments*	
Rifaximin		Osmotic laxative Acidification of the colon ↓ Urease-producing bacteria ↓ Ammonia production ↓ Ammonia absorption		Improved cognitive function, driving performance Cost-effective in preventing accidents		Most extensively studied ↓progression to overt HE		↓ likelihood of overt HE		Mainstay of HE treatment and prophylaxis Cost-effective	
Rifaximin		↓ Urease-producing bacteria ↓ Ammonia production		Improves cognitive function, QOL		↓ breakthrough HE, hospitalization		Not studied		Modulates flora Does not cause resistance	
Probiotics		Improve dysbiosis		Improvement in cognitive tests Improved QOL ↓ endotoxins		↓ risk of hospitalization		Not studied		Well tolerated Available without prescription	
BCAA		Promotes the synthesis of glutamine from ammonia in skeletal muscle		Unclear		Improves recurrent HE		Not studied		No effect on overall mortality	
LOLA		Ammonia scavenging - ↑ production of urea in hepatocytes, activating glutamine synthase in hepatocytes and skeletal muscle		No improvement in covert HE ↑ progression to overt HE		↑ progression to overt HE		Not studied		Evidence conflicting except in overt HE, more studies needed	
Glycerol phenylbutyrate		↑ Excretion of glutamine		Not studied		↑ time to recurrence		Not studied		No benefit in patients on rifaximin	
Zinc		If deficient reduce urea cycle utilization of ammonia		Improvement in cognitive tests		None		Not studied		No evidence on other outcomes	

Adapted and modified from Rathi and Dhiman^31^

Rifaximin

Rifaximin is a semisynthetic, nonabsorbable gut-specific antibiotic. It modulates the gut microbiota and reduces the harmful bacteria. Prolonged use can induce antimicrobial resistance. Bass et al^21^ showed that rifaxi-min reduced the episodes of breakthrough HE when compared with placebo (22.1 *vs* 45.9%) over a period of 6 months in patients with chronic liver disease. Rifaxi-min has a definite role in treatment of HE. In secondary prophylaxis, data suggest its use after second episode of HE (breakthrough episode).

Probiotics

The probiotics are a new emerging therapy in HE. Pro-biotics modulate the number and composition of gut microbiota and, therefore, affect the gut brain axis. They reduce the gut-derived toxins and, hence, the ammonia levels. We have previously showed that in patients with cirrhosis who previously had HE there was a significant decline in hospitalizations and complications of cirrhosis, with improvement in the Child-Turcotte-Pugh score and model for end-stage liver disease in probiotic group as compared with placebo.^22^ A recent systemic review on probiotics in HE has shown that use of probiotics reverses MHE and reduces the development of overt HE as compared with placebo; however, no benefits were found when compared with standard therapy for HE like lactulose, rifaximin, and L-ornithine-L-aspartate (LOLA).^23^

L-ornithine-L-aspartate

It is a salt of amino acids ornithine and aspartate that helps in urea cycle metabolism and clearance of ammonia. Stauch et al^24^ showed improvement in the number connection test and blood ammonia level with the use of LOLA as compared with placebo in overt HE. The data for LOLA are still conflicting.

**Flow Chart 1:**
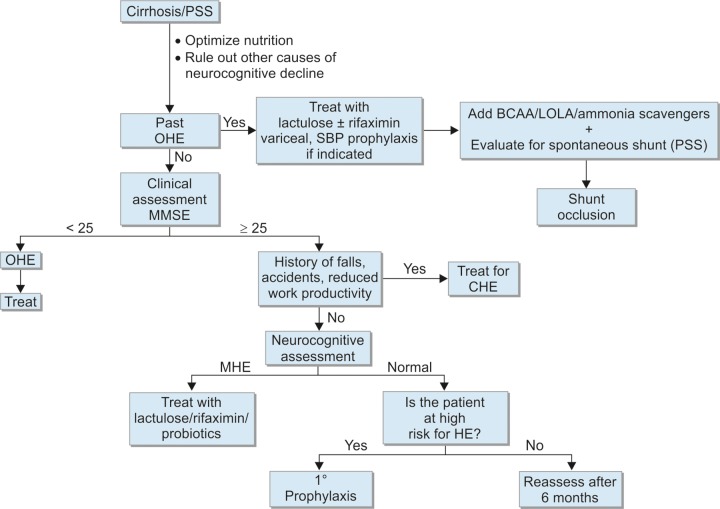
Algorithm for outpatient management of HE; OHE: Overt HE; PSS: Portosystemic shunt. Adapted and modified from Rathi and Dhiman^31^

Nutrition

It is very important to understand that cirrhosis is a state of malnutrition. Ammonia clearance occurs through the liver as well as muscle. The sarcopenia associated with cirrhosis leads to reduced ammonia clearance and precipitates HE. Therefore, nutrition is an important target in cirrhosis. The ISHEN recommends 1.2 to 1.5 gm/kg/ day protein and 35 to 40 kcal/kg/day in cirrhosis. It is preferable to take dairy and vegetable proteins. Patients should be encouraged to consume small, frequent meals. A late night snack rich in energy is helpful in curtailing catabolism due to fasting.^25^ Also, there are data about breakfast improving the cognition. There are data for use of branched chain amino acids in HE; however, long-term efficacy with its use is conflicting.

Shunt Occlusion

Spontaneous portosystemic shunts, which are enough to divert ammonia directly to systemic circulation, put the patient at risk of developing encephalopathy. Balloon retrograde transvenous occlusion of shunts has shown improvement in symptoms and survival benefits.^26^ Patients with TIPSS and HE may need revision of shunt and reduction in diameter.

Liver Transplant

In recurrent and chronic persistent HE, with deteriorating liver functions, the medical therapy fails to improve the outcome. Only liver transplant in this situation can help to salvage the patient and improve the outcome.

## TREATMENT OF COVERT HE

Covert HE includes both MHE and grade I HE. Although there are no definite guidelines to treat covert HE, various studies have shown efficacy of different drugs. We showed lactulose therapy to be effective in improving cognitive function and health-related quality of life (HRQOL) in patients of cirrhosis with MHE after 3 months of therapy.^27^ In another study by Sidhu et al,^28^ rifaximin use as compared with placebo showed improvement in cognitive function and HRQOL in patients of cirrhosis with MHE. In chronic persistent subclinical HE, LOLA has shown to improve the psychometric test performance and reduce the blood ammonia levels as compared with placebo in individual studies;^29^ more data are needed for its use. However, there is a need for more data before a clear recommendation about MHE treatment can be made.

We performed a network meta-analysis,^30^ which included 27 randomized controlled trials (RCTs) for reversal of minimal HE and 21 RCTs to prevent development of overt HE. For reversal of minimal HE, rifaximin followed by lactulose, probiotics/lactulose combination, LOLA, prebiotic/probiotic/symbiotic (PPS) combination, and branched chain amino acids (BCAA) were found to be better than placebo or no drug respectively. For prevention of development of overt HE, lactulose, LOLA, and PPS combination was found to be superior to placebo or no intervention. However, rifaximin was not found to be significant in prevention of overt HE. Lactulose is the only intervention found useful in both reversal of minimal HE and prevention of development of overt HE.

## PRIMARY PROPHYLAXIS

Routine use of drugs to prevent episode of overt HE is not recommended. However, cirrhosis with decompensation having high risk of development of HE may be treated with lactulose for primary prophylaxis of overt HE.

## CONCLUSION

In cirrhosis of liver, HE is a complication, which needs to be addressed immediately, as it has impact on morbidity as well as mortality. Overt HE reduces the work performance, decreases work productivity, impairs the driving ability, and increases the chances of accidental injuries on complex machinery. Its diagnosis through psychometric tests and newer computerized tests is imperative. The role of newer biomarkers and imaging modalities is still in evolution. The lactulose, rifaximin, and probiotics have enough data to consider them as mainstay of therapy. The early stages of encephalopathy are manageable; however, higher grades of HE (grades III and IV) are associated with high mortality. The role of systemic shunting and neuroinflammation needs to be explored further to evaluate newer targets of therapy. In recurrent and persistent HE, only liver transplant can change the natural history of cirrhosis. Finally, lactulose is effective both in improving reversal of minimal HE and in reducing the risk of development of overt HE.
